# A Comparative Study of Almond Biodiesel-Diesel Blends for Diesel Engine in Terms of Performance and Emissions

**DOI:** 10.1155/2015/529808

**Published:** 2015-03-22

**Authors:** Nidal H. Abu-Hamdeh, Khaled A. Alnefaie

**Affiliations:** Mechanical Engineering Department, Faculty of Engineering, King Abdulaziz University, P.O. Box 80204, Jeddah 21589, Saudi Arabia

## Abstract

This paper investigates the opportunity of using almond oil as a renewable and alternative fuel source. Different fuel blends containing 10, 30, and 50% almond biodiesel (B10, B30, and B50) with diesel fuel (B0) were prepared and the influence of these blends on emissions and some performance parameters under various load conditions were inspected using a diesel engine. Measured engine performance parameters have generally shown a slight increase in exhaust gas temperature and in brake specific fuel consumption and a slight decrease in brake thermal efficiency. Gases investigated were carbon monoxide (CO) and oxides of nitrogen (NO_*x*_). Furthermore, the concentration of the total particulate and the unburned fuel emissions in the exhaust gas were tested. A blend of almond biodiesel with diesel fuel gradually reduced the engine CO and total particulate emissions compared to diesel fuel alone. This reduction increased with more almond biodiesel blended into the fuel. Finally, a slight increase in engine NO_*x*_ using blends of almond biodiesel was measured.

## 1. Introduction

Current civilization is greatly reliant on fossil energy. Fossil fuels are the greatest energy source among all energy resources. The major part of energy requirements in the world is provided through petroleum resources such as natural gas, oil, and coal. Fossil fuel depletion is to increase with time. Since fossil resources are nonrenewable, rising demands and diminishing supplies keep fuel price rising dramatically.

The use of diesel engines resulted in numerous mechanized improvements for decreasing pollutant emissions as well as fuel usage [1]. Diesel machines are widely used in heavy-duty applications especially in the construction and farming sectors. Accordingly, the rate of reduction of diesel fuel is the greatest among gasoline fuels, which subsequently results in a greater rate of price increase of diesel fuel than other gasoline fuels. Additionally, the growing concern about environmental pollution since the 1990s has boosted the interest in alternative fuels. This has led to more financial support for research studies in energy management and conservation. Recently, the issues of steadily rising fuel prices, declining oil storage, and air contamination have resulted in the investigation of fossil fuel alternatives. These issues added to the increase in greenhouse gases such as CO_2_ which is causing climate change and global warming have robustly boosted the interest in making use of biodiesel for power generation. Biodiesel is an expression usually used to refer to fatty acid methyl esters that are often created from animal fats or extracted from vegetables and have acceptable capabilities to be used in diesel engines. Because diesel fuel and vegetable oils have close cetane numbers, biodiesel made from vegetable oils might be used in current diesel engines after minor alterations [2–4].

Several studies have investigated the use of vegetable oils as alternative fuels [5–9]. Some of these research studies [10–16] reveal that there is little harm including lubricating oil thickening, injector coking, and gum formation ring sticking. The nonvolatility and excessive stickiness of pure vegetable oils were the major causes of these problems [17]. Talebi et al. [18] reported on a new software package, the BiodieselAnalyzer, for predicting the properties of a prospective biodiesel. Such software can estimate 16 different quality parameters of a biodiesel based on the fatty acid methyl ester profile of the oil feedstock used in making it.

Almonds (*Prunus dulcis*) are believed to be the most widely spread among tree nuts all over the world and are top of the list in tree nut output. They are affiliated to the Rosaceae group that also contains pears, prunes, and apples [19]. Almonds are widely produced in areas characterized by a Mediterranean climate [20], including many countries in the Mediterranean, all Middle East countries, and some countries in the Southern Hemisphere. The biggest producer of almonds in the world is the United States, specifically California; as a result, almond oil is mostly produced there [21]. Because biofuels are made from renewable sources, developing the technology to produce them must ensure the adequate supply of transporting fuel in the future as well as providing assurance against the uncertainty surrounding the petroleum resource timeline. Almond oil could be extracted from the seeds which contain a high percentage of oil [22–25]. As far as the author knows, no investigations were done on the employment of almond oil as a substitute for diesel fuel. Therefore, this study provides options for new valuable use for an existing crop.

Diesel engines are main sources of environmental pollutants such as carbon monoxide (CO), carbon dioxide (CO_2_), oxides of nitrogen (NO_*x*_), and partly burned (or burned) hydrocarbons (HC) organic compounds [26–29]. Usually, the portion of the fuel drawn in is not enough to hold great consequence on effectiveness but could be sufficient to cause severe air contamination. Such emissions have always been a critical issue in air pollution [30–32]. Engine exhaust emissions usually contain nitrogen oxides. As Lapuerta et al. [33] showed in their review paper, biodiesel exhibits an increase in NO_*x*_ concentration compared to diesel and only a few studies showed a percentage drop-off in NO_*x*_ concentration [34, 35].

This work aims to compare the various performance parameters and emissions of a single-cylinder diesel engine operating on almond biodiesel with an engine operating on pure diesel fuel through laboratory measurements in terms of exhaust gas temperature, brake specific fuel consumption, and brake thermal efficiency. Emissions investigated were carbon monoxide (CO), oxides of nitrogen (NO_*x*_), and concentration of the total particulate and the unburned fuel emissions in the exhaust gas.

## 2. Materials and Methods

### 2.1. Extraction and Transesterification of Almond Oil

After peeling the almond seeds, they were dried at nearly 30°C and then crushed in a blender. Powdered seeds were kept at 5°C in polyethylene bags before analysis. The Bligh-Dyer method was used to extract almond oil [36]. Ground seeds were harmonized with a chloroform-methanol (CHCl_3_/MeOH) mixture (1 : 1) and water. Two phases were obtained, aqueous layer (MeOH-water) and organic layer (CHCl_3_). A rotary evaporator was used for evaporating off the solvent (CHCl_3_) for the recovery of oil. A residual solvent was detached by oven drying for 1 hour at 60°C and flushing with 99.9% nitrogen. The transesterification of almond oil was performed as given by Hossain et al. [37] to guarantee fewer impurities.

### 2.2. Properties of Almond Biodiesel and Its Blends

The selected engine fuel was a local commercially available diesel fuel. A laboratory preparation of blends of almond biodiesel with diesel fuel was performed to operate a diesel engine and to make measurements of emissions and performance parameters. The ratios of blends selected were B0, B10, B30, and B50 on volume basis of almond biodiesel in an almond biodiesel-diesel fuel mixture. They are referred to as B0 (0% almond biodiesel-100% diesel fuel), B10 (10% almond biodiesel-90% diesel fuel), B30 (30% almond biodiesel-70% diesel fuel), and B50 (50% almond biodiesel-50% diesel fuel), respectively. These abbreviations are used throughout the current study.

Experimental measurements of the chemical and physical properties of the almond biodiesel with diesel fuel and diesel fuel alone have been performed since they directly affect emissions, fuel droplet dimension, and spray features. The analysis procedures and complete details have been followed as described in Kannan et al. [38]. The measured properties of diesel, biodiesel from almond oil, and different ratios of their blends according to ASTM standard are shown in Table 1.

### 2.3. Procedure and Experimental Setup

**Figure 1:**
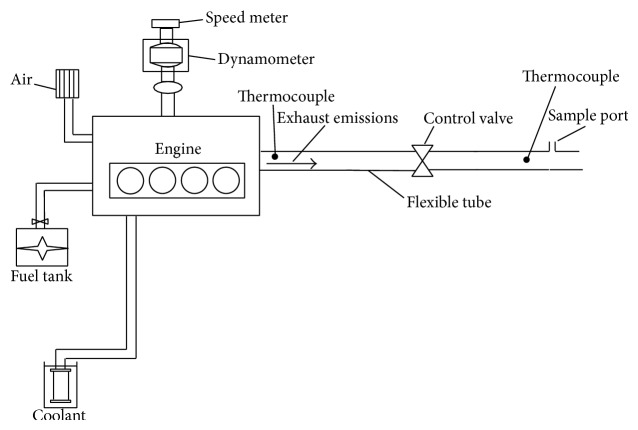
Schematic arrangement of the system.

Experiments were performed to study biodiesel from almond oil as a substitute fuel to operate a diesel engine and the performance data were recorded. The exhaust gases constitution and the percentage of contaminant emissions were also measured and investigated. The experimental setup, schematically shown in Figure 1, consists of a single-cylinder, water-cooled, naturally aspirated, direct-injection (DI), and variable compression engine mounted on a standard TEQUIPMENT TD 43 test rig made in Britain. Swept volume of the engine was 583 cm^3^ with a 95 mm bore and 82 mm bore by stroke. The injection system consists of an in-line fuel injection pump and throttle-type nozzle. The combustion chamber is direct injection type with a bowl-in piston design. The injection timing and injection pressure were set at 21° crank angle bTDC and 20 MPa, respectively. The cylinder pressure at each crank angle was measured and stored by a digital data acquisition system. It consisted of a Kistler water-cooled flush mounted piezoelectric pressure transducer in conjunction with Kistler charge amplifier for converting the electric charge into voltage. It could measure and store up to 200 cycles engine pressure histories. The measured data can be analyzed online or stored for postprocessing. A Chromel-Alumel (k-type) thermocouple together with a calibrated digital display was used to measure exhaust gas temperature. Load was applied through the engine's connection to an electrical generator dynamometer and could be varied by changing the control panel voltage. A rotameter was used to measure the flow rate of cooling water. The engine was similar to an engine used in a former study [39].

The differences in the measured performance and exhaust emission parameters from the “baseline” operation of the engine and all fuels tested were determined and compared. The experimental work started with a preliminary investigation of the engine running on neat diesel fuel, to determine the engine's operating characteristics and exhaust emission levels constituting the “baseline.” The data gathering was made at five engine torques from 4 to 20 N · m at an increment of 4 N · m each time. A control switch on the test rig was used to vary the engine torque. The time of injection was set at 21° bTDC and kept the same during all tests. In addition, the compression ratio was maintained at 18 : 1. The engine was warmed up at no load for 15 minutes to reach steady state conditions in all experiments. The speed of the engine was measured using a tachometer and it was adjusted to the rated speed of 1500 rpm by adjusting the governor connected to the fuel pump. A constant speed of 1500 rpm for the engine was used throughout this study. The same procedure was repeated for each fuel blend by repeating the same operating process for each fuel blend maintaining the same operating conditions. Before the start of the experiment with new fuel variant, the fuel used in the previous experiment was completely purged from the fuel line, filter, fuel pump, and fuel tank and the engine was left to operate for about three minutes to stabilize in its new condition. The data collected from the experimental tests included brake torque, exhaust and coolant temperatures, emissions from exhaust including carbon monoxide and nitrogen oxides, and emissions of total particulate. Engine torque, brake power, various temperatures, and air flow rate readings were taken from their meters on the test rig. Inlet and outlet cooling water temperatures were measured and recorded. Gas samples were drawn from the engine exhaust for the analysis of the contaminants.

For measuring the oxides of nitrogen (NO_*x*_), carbon monoxide (CO), and total particulate emissions in the exhaust, a gas phase analysis scheme depicted a suggestive amount of the exhaust from the tester port. Sampling procedures and analysis performed were followed as in Abu-Hamdeh [39] in the fact that samples of the exhaust gas were taken from the manifold of the exhaust and supplied to the analyzing instruments. NO_*x*_ measurements were obtained using Thermoelectron Model 10 AR chemiluminescent analyzer, while the samples of the exhaust were fed through a moisture trap cooled by ice and fed through a particulate filter before entering the gas analyzer. This was to ensure full drying and filtering of the gases before starting the analysis process. A gas analyzer (MSI 2000) was utilized to capture samples and evaluate the concentrations of CO in the exhaust gases. A gas extractor probe was used to remove the flue gas from the flue while measuring its temperature via a temperature sensor attached to its end. A suction pump, controlled electronically, was used to pump a constant flow rate of the gas into the evaluating compartment. After feeding throughout a constraint, same amounts of gas were pointed in the direction of electrochemical sensors via dosage compartment.

For the collection of particulate matter, unloaded clean strainers were inserted in an oven for about 24 h at 220°C. A calibrated sensitive digital balance (accuracy of ±0.02 mg) was used to weigh the strainers. The collected samples were passed out of the strainer for 3–5 min, allowing the collection of 20–90 mg of particulate matter. When the sampling process finished, the charged strainer was carefully taken off the probe. The strainer was equilibrated for a minimum of 24 hours in a controlled environment prior to being weighed for total particulate matter mass. During the equilibration period, relative humidity was maintained at a mean value of 35–40% and air temperature at a mean of 21–23°C. The particulate matter's weight was found out from the difference between the final weight of the strainer and its initial weight. The results obtained from the instruments mentioned above, accompanied by the engine torque and speed, were supplied to a data acquisition system. Each reported value for all measured parameters is the average of three replicates.

## 3. Results and Discussion

Density is an important property of fuel for compression ignition engines. It is worth noting that fuel density increases with the increase in the blending percentage. The 50% and 100% almond biodiesel blends have about 2.6% and 7.4% higher density, respectively, than diesel fuel. Preheating of biodiesel before injection could be done to overcome the problem of higher fuel density by taking advantage of the high temperature of the engine exhaust gas. The kinematic viscosity was measured for biodiesel from almond oil and was found to be 4.726 cSt, which is about 40% more than the kinematic viscosity of diesel (3.384 cSt). A decrease in the blending percentage of almond biodiesel decreased the kinematic viscosity of the mixture. The shape of the fuel droplets and atomization are affected by the fuel viscosity. Higher viscosity of fuel may cause problems and smoky exhaust. This requires higher spraying pressure to obtain the desired spray pattern inside the cylinder. In contrast, very low viscous fuel would prevent accurate metering of the fuel especially in older engines due to the leakage from piston walls of the injection pump [37]. Preheating of biodiesel before injection either in the fuel tank or in the fuel lines could be done to overcome the problem of higher viscosity of biodiesel oils by taking advantage of the high temperature of the engine exhaust gas. The heating value of the almond biodiesel was measured and found to be 41.761 MJ/kg, which is 14% lower than the heating value of diesel. The flash point of almond biodiesel was 145°C while diesel has a flash point of 68°C. Transportation and safe storage are improved by higher flash point. Blending of almond biodiesel with diesel fuel reduced the value of the flash point. Still the flash point of the diesel fuel is comparatively lower than that of the different ratios of biodiesel blends. Diesel fuel and pure almond biodiesel have about equal values of carbon and hydrogen content, but in terms of ash diesel fuel contains higher percentage than almond biodiesel.

**Figure 2:**
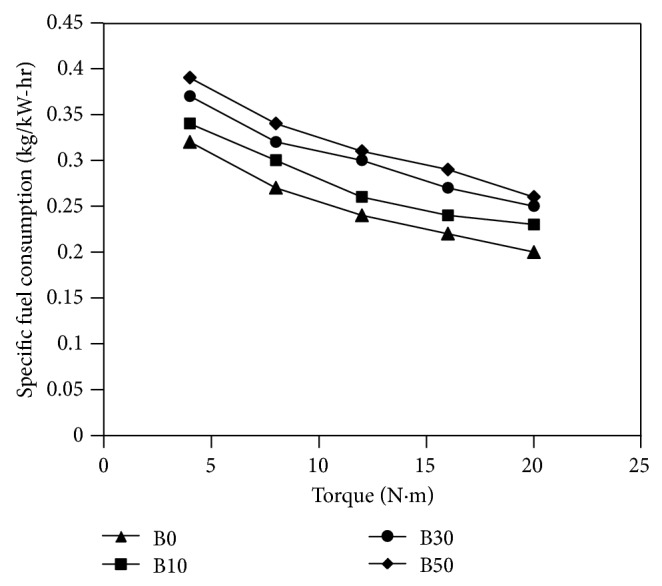
Variations of specific fuel consumption versus measured torque for fuels used in the study.

Experimental investigations of almond biodiesel on single-cylinder diesel engine were done and several parameters, such as specific fuel consumption (bsfc), brake thermal efficiency (*η*_*b*_), exhaust gas temperature (*T*_*g*_), carbon monoxide (CO), nitrous oxide (NO_*x*_), particulate matter, and unburned fuel emissions, have been determined. The results obtained are shown in Figures 2 through 8.

Figure 2 shows the effect of blending ratio and measured torque on the specific fuel consumption (bsfc) at 1500 rpm. Blends of biodiesel in general have more specific fuel consumption than diesel fuel at the same torque and it increases with increasing blending ratio of biodiesel. The first reason for that is the different LHV of the biodiesel with respect to the fossil diesel. Other secondary reasons are poor atomization and the formation of mixtures with biodiesel blends because of their higher density and kinematic viscosity compared to their values with diesel fuel. That is to say, more percentages of biodiesel blends are needed to produce the same torque.

**Figure 3:**
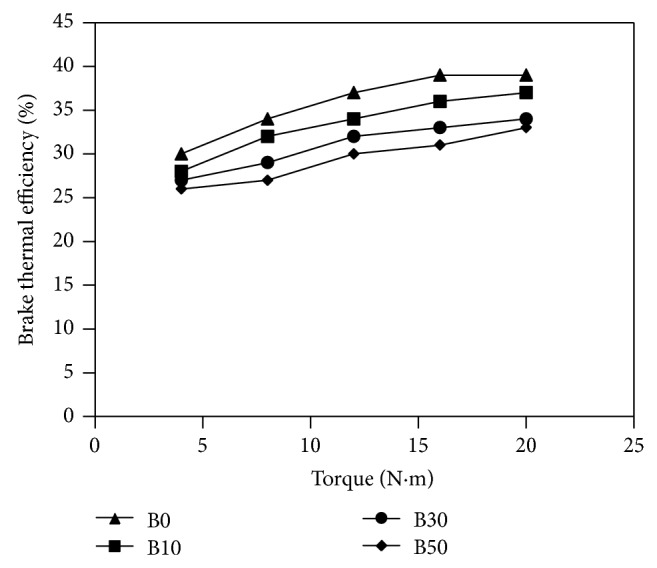
Variations of brake thermal efficiency versus measured torque for fuels used in the study.

Figure 3 illustrates the change in the brake thermal efficiency (*η*_*b*_) as a function of torque measured. The figure shows that for a given torque the thermal efficiency for biodiesel blends is less than thermal efficiency for diesel fuel. A major reason for this tendency is the higher density and viscosity of biodiesel in comparison to diesel fuel which results in poor atomization and leads to lower combustion efficiency especially at higher torques. The thermal efficiency improvement of the 10% almond biodiesel blend over the other blending ratios can be accredited to the better oxygen content which enhances combustion particularly during the stage of diffusion combustion and to the reduction of the friction loss because of higher lubricity of almond biodiesel. Further reduction in brake thermal efficiency with increasing the percentages of almond biodiesel blends can be noticed. This reduction in thermal efficiency with increasing the percentages of almond biodiesel blends can be credited to the inferior combustion characteristics of the blends due to their relatively poor volatility, high viscosity, lower calorific values, and slow burning rate and that overcomes the surplus oxygen existing in the almond biodiesel.

**Figure 4:**
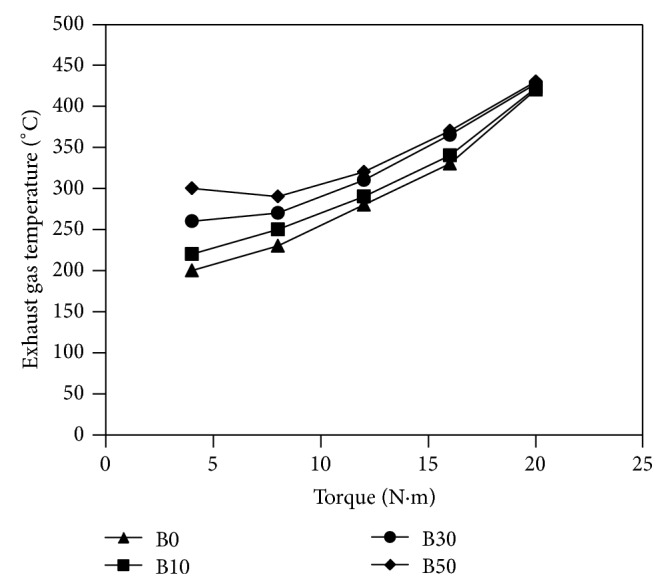
Variations of exhaust gas temperature versus measured torque for fuels used in the study.

Figure 4 illustrates the change of exhaust gas temperature (*T*_*g*_) with measured torque for the fuels used. Diesel fuel has lower exhaust gas temperature than biodiesel blends, and it increases with the blending ratio. This could mean earlier burning in case of higher blends of biodiesel due to shorter premixed combustion period. High cetane number values reduce the premixing time and move the combustion phasing earlier in the compression stroke. At higher loads, biodiesel blends and diesel fuel have comparable exhaust gas temperature.

**Figure 5:**
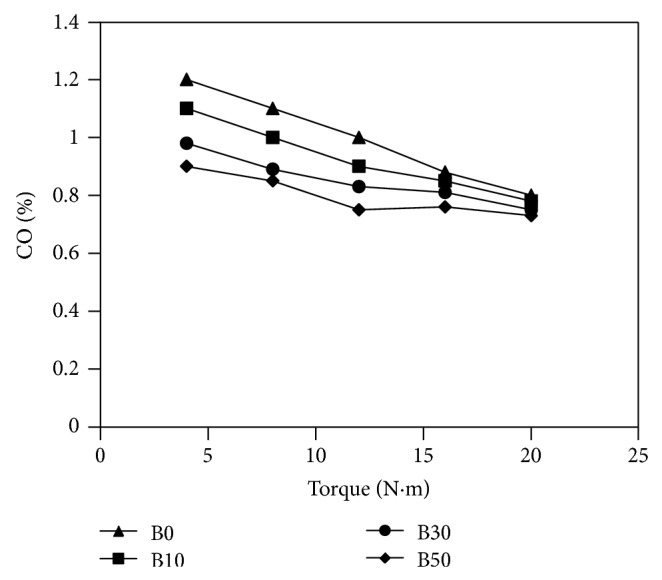
Variations of carbon monoxide concentration in the exhaust versus measured torque for fuels used in the study.

The concentration change of carbon monoxide (CO) with respect to measured torque is illustrated in Figure 5. The first observation that can be noticed in the figure is that CO emissions decrease with increasing measured torque. In all the internal combustion engines upon raising the load (and then the torque) at constant engine speed there is a significant increment of combustion temperature with remarkable conversion of CO to CO_2_ and then less emission of CO at the exhaust. Furthermore, it is observed that the higher biodiesel blend value, the lower CO emissions in the exhaust gas at any measured torque. For the same load, it seems that blends of biodiesel accelerate the reaction rates and propagate the flame successfully throughout the fuel mixture resulting in improved combustion. This is a result of the existence of more oxygen content in biodiesel. This probably decreased the CO in the exhaust gas.

**Figure 6:**
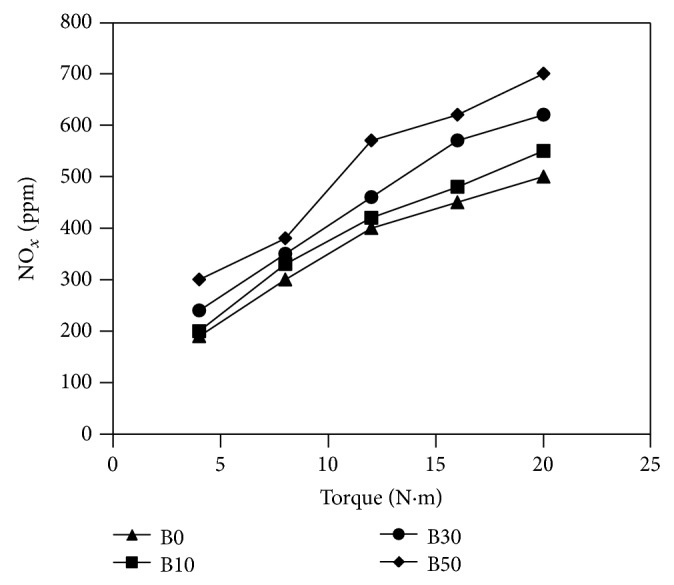
Variations of NO_*x*_ concentration in the exhaust versus measured torque for fuels used in the study.

The variation of NO_*x*_ percentage in the exhaust gas as a function of torque measured is shown in Figure 6. NO_*x*_ concentration in all blends shows higher values compared to diesel fuel. The higher blends of biodiesel produced higher NO_*x*_ emissions. The production of NO_*x*_ is influenced by maximum temperature of the cylinder charge. Generally, the existence of oxygen in the biodiesel molecules performs as an extra factor to increase NO_*x*_ making. At higher loads, enhanced combustion and higher flame temperature increase NO_*x*_ emissions. Moreover, NO_*x*_ emissions are noticed to rise with the increase of oxygen content in the fuel. This is due to the local leaner air fuel ratio as the almond blends contain some oxygen in their molecular structure.

**Figure 7:**
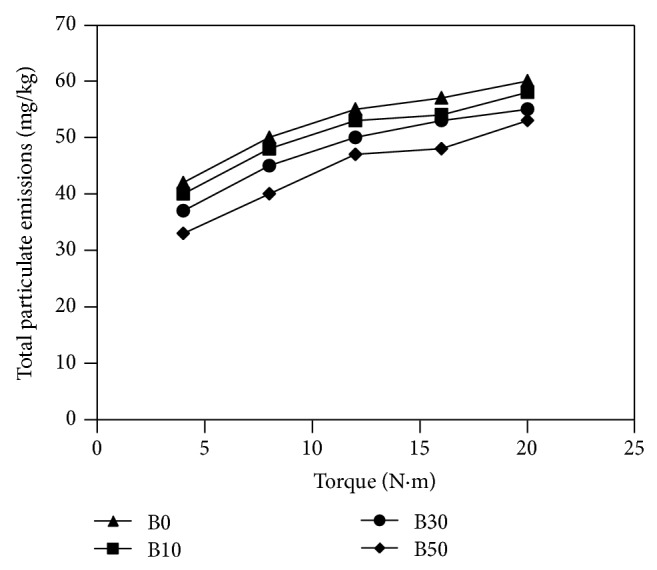
Total particulate emissions versus measured torque for fuels used in the study.

**Figure 8:**
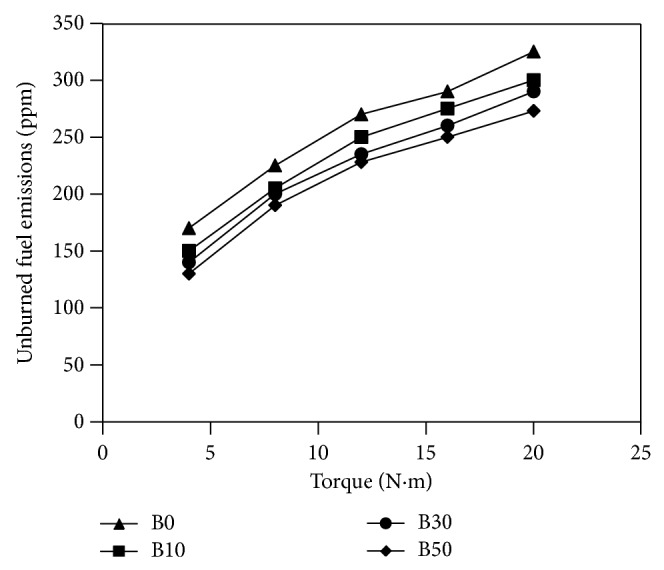
Unburned fuel emissions versus measured torque for fuels used in the study.

A glimpse at the curves in Figure 7 shows that the percentage of biodiesel blend influenced the concentration of particulate matter in the exhaust. Agglomerates of tiny carbonaceous particles are the primarily comprise of particulate emissions from diesel engines. The particulate matter character is highly vassal to the extent to which combustion progresses as preblend combustion in opposition to propagation of combustion flame [40]. Figure 7 illustrates the change in particulate emissions for different fuels used with torque measured. The particulate matter concentration was obtained as the ratio of the particulates' mass in milligrams collected by the filter from the exhaust stream to the total mass of the exhaust in kilograms passing through the filter in the same interval of time. Figure 7 points out that the increased load led to an increase in the particulate emissions. It could be read from the same figure that at any torque value there is an inverse relationship between the biodiesel blending ratio and the particulate emissions; that is, when biodiesel blending ratio increases the particulate emissions decrease. The 50% biodiesel blend produced the lowest particulate emissions compared to all fuels used in the study.  Figure 8 shows the variation of unburned fuel emissions for different fuels used with torque measured. The 50% biodiesel blend produced the lowest unburned fuel emissions compared to all the test fuels. At full load conditions 50% biodiesel blend emitted 18% lower unburned fuel emissions than that of diesel fuel. At the same load, the B10 and B30 blends produced 6% and 10% lower unburned fuel emissions than diesel fuel, respectively. This shows that the particulate and unburned fuel emissions tend to reduce as the content of oxygen of the fuel increases, which leads to the particulate oxidation through the combustion interval. It is worth mentioning that the flow of the exhaust stream through oxidation catalysts reduces particulate emissions.

## 4. Conclusion

The emissions and performance features of a single-cylinder, naturally aspirated, diesel engine fueled with diesel-almond biodiesel blends were investigated in this study. Blends used were 10%, 30%, and 50% of almond biodiesel with diesel fuel. The blends and the diesel fuel were examined under various load conditions. In terms of engine performance, it was found that increasing blending ratios of almond biodiesel increased the specific fuel consumption (bsfc) for biodiesel and exhaust gas temperature (*T*_*g*_); on the other hand it decreased brake thermal efficiency (*η*_*b*_). Blending ratio B10 has the minimum specific fuel consumption and the maximum brake thermal efficiency among other blend ratios. In terms of emissions, it was found that increasing blending ratios of almond biodiesel decreased the particulate matter, unburned fuel emissions, and CO content in the exhaust gas. However, higher blends of biodiesel produced higher NO_*x*_ emissions. In general, it is feasible to use biodiesel made from almond oil blends to run a diesel engine but more investigation is required to reach clear-cut conclusions and to give more details on the potential of this biomass as a fuel.

## Figures and Tables

**Table 1:** Physical and chemical specifications of fuels.

Property	Test method	100% diesel	30% almond biodiesel-70% diesel	50% almond biodiesel-50% diesel	100% almond biodiesel
Flash point (°C)	ASTM D 93	68	80	86	145
Fire point (°C)	ASTM D 93	82	97	102	154
Ash content (% by weight)	ASTM D 482	0.017	0.015	0.014	0.013
Kinematic viscosity at 40°C (cSt)	ASTM D 445	3.384	3.731	4.235	4.726
Density at 25°C (g/cm^3^)	ASTM D 1298	0.848	0.856	0.870	0.911
Oxygen content (% vol.)		0	4.83	9.67	12.6
Carbon content (% by mass)	ASTM D 3176	80.6	80.8	81.0	81.1
Hydrogen content (% by mass)	ASTM D 3176	5.4	5.2	5.2	5.1
Calorific value (MJ/kg)	ASTM D 5865	48.610	44.727	43.532	41.761
Cetane number	ASTM D 613	47.48	48.31	52.54	54.32
